# Influence of Multidrug Resistance-Associated Proteins on the Excretion of the ABCC1 Imaging Probe 6-Bromo-7-[^11^C]Methylpurine in Mice

**DOI:** 10.1007/s11307-018-1230-y

**Published:** 2018-06-25

**Authors:** Viktoria Zoufal, Severin Mairinger, Markus Krohn, Thomas Wanek, Thomas Filip, Michael Sauberer, Johann Stanek, Alexander Traxl, John D. Schuetz, Claudia Kuntner, Jens Pahnke, Oliver Langer

**Affiliations:** 1 Biomedical Systems, Center for Health & Bioresources, AIT Austrian Institute of Technology GmbH, Seibersdorf, Austria; 2 Department of Neuro-/Pathology, University of Oslo (UiO) and Oslo University Hospital (OUS), Oslo, Norway; 3 Department of Clinical Pharmacology, Medical University of Vienna, Vienna, Austria; 4 Department of Pharmaceutical Sciences, St. Jude Children’s Research Hospital, Memphis, TN USA; 5 LIED, University of Lübeck, Lübeck, Germany; 6 Leibniz-Institute of Plant Biochemistry, Halle, Germany; 7 Division of Nuclear Medicine, Department of Biomedical Imaging and Image-guided Therapy, Medical University of Vienna, Vienna, Austria

**Keywords:** PET, 6-Bromo-7-[^11^C]methylpurine, ABC transporters, Multidrug resistance-associated proteins, Renal excretion, Glutathione-S-transferases (GST)

## Abstract

**Purpose:**

Multidrug resistance-associated proteins (MRPs) mediate the hepatobiliary and renal excretion of many drugs and drug conjugates. The positron emission tomography (PET) tracer 6-bromo-7-[^11^C]methylpurine is rapidly converted in tissues by glutathione-*S*-transferases into its glutathione conjugate, and has been used to measure the activity of Abcc1 in the brain and the lungs of mice. Aim of this work was to investigate if the activity of MRPs in excretory organs can be measured with 6-bromo-7-[^11^C]methylpurine.

**Procedures:**

We performed PET scans with 6-bromo-7-[^11^C]methylpurine in groups of wild-type, *Abcc4*^(−/−)^ and *Abcc1*^(−/−)^ mice, with and without pre-treatment with the prototypical MRP inhibitor MK571.

**Results:**

6-Bromo-7-[^11^C]methylpurine-derived radioactivity predominantly underwent renal excretion. In blood, MK571 treatment led to a significant increase in the AUC and a decrease in the elimination rate constant of radioactivity (*k*_elimination,blood_). In the kidneys, there were significant decreases in the rate constant for radioactivity uptake from the blood (*k*_uptake,kidney_), *k*_elimination,kidney_, and the rate constant for tubular secretion of radioactivity (*k*_urine_). Experiments in *Abcc4*^(−/−)^ mice indicated that Abcc4 contributed to renal excretion of 6-bromo-7-[^11^C]methylpurine-derived radioactivity.

**Conclusions:**

Our data suggest that 6-bromo-7-[^11^C]methylpurine may be useful to assess the activity of MRPs in the kidneys as well as in other organs (brain, lungs), although further work is needed to identify the MRP subtypes involved in the disposition of 6-bromo-7-[^11^C]methylpurine-derived radioactivity.

**Electronic supplementary material:**

The online version of this article (10.1007/s11307-018-1230-y) contains supplementary material, which is available to authorized users.

## Introduction

Adenosine triphosphate-binding cassette (ABC) and solute carrier (SLC) transporters play a pivotal role in controlling absorption, tissue distribution, and excretion of diverse drugs and drug metabolites [1, 2]. Changes in transporter activities, due to drug-drug interactions, genetic polymorphisms, or disease, can sometimes lead to pronounced changes in drug pharmacokinetics. These changes may critically affect drug safety and efficacy. Regulatory authorities therefore request that the interaction of new drug candidates with ABC and SLC transporters should be assessed during drug development [3, 4]. Consequently, there is a great interest to characterize the influence of ABC and SLC transporters on drug disposition both in preclinical species and in humans. The non-invasive nuclear imaging method positron emission tomography (PET) has emerged as a promising technique to assess *in vivo* if drugs interact with ABC and SLC transporters [5–8]. This can either be done by studying the effect of unlabeled drugs on the disposition of radiolabeled probe substrates, which are transported by the transporters of interest, or by directly studying the disposition of the radiolabeled drug of interest [5]. The utility of PET to study drug transporters critically depends on the availability of suitable transporter-selective radiolabeled probe substrates. Several effective PET probe substrates have been described for assessing the activity of P-glycoprotein (humans: ABCB1, rodents: Abcb1a/b) and breast cancer resistance protein (humans: ABCG2, rodents: Abcg2) [9]. On the other hand, there is currently a scarcity of PET tracers, which allow for measuring the activities of multidrug resistance-associated proteins (MRPs), such as MRP1, 2, 3, and 4 (humans: ABCC1-4, rodents: Abcc1-4). MRPs are involved in the tissue distribution as well as in the hepatobiliary and renal excretion of many anionic substrates, including drugs and drug conjugates [10]. While one PET probe substrate ([^11^C]dehydropravastatin) was found suitable to measure the activity of Abcc2 in the rat liver [11], no PET tracers are, to our knowledge, currently available for measuring the activity of MRPs in the kidneys. 6-Bromo-7-[^11^C]methylpurine has been used before to measure the activity of Abcc1 in the brain and in the lungs of mice [12, 13]. This radiotracer is a prodrug, which is taken up into tissue, presumably by passive diffusion, where it is converted by glutathione-*S*-transferases into its glutathione conjugate *S*-(6-(7-[^11^C]methylpurinyl))glutathione, which is an ABCC1 substrate [14]. Okamura et al. have shown that 6-bromo-7-[^11^C]methylpurine is almost quantitatively converted into its glutathione conjugate in the brain and the lungs of mice within 15 min after intravenous (i.v.) injection [12, 13]. Moreover, they found that the elimination rate constant (*k*_elimination_) of radioactivity is reduced by 9.3- and 17.2-fold in the brain and the lungs, respectively, of *Abcc1*^(−/−)^ mice, relative to wild-type mice.

The aim of the present study was to extend the utility of 6-bromo-7-[^11^C]methylpurine to measure the activities of MRPs in excretory organs by studying whole-body distribution of 6-bromo-7-[^11^C]methylpurine-derived radioactivity with PET imaging in wild-type, *Abcc4*^(−/−)^, and *Abcc1*^(−/−)^ mice, with and without pre-treatment with the prototypical MRP inhibitor MK571.

## Material and Methods

### Chemicals

Unless otherwise stated, all chemicals were purchased from Sigma-Aldrich (Schnelldorf, Germany) or Merck (Darmstadt, Germany). The MRP inhibitor MK571 was obtained from Santa Cruz Biotechnology (Dallas, TX, USA). Before each administration, MK571 was dissolved in phosphate-buffered saline (PBS) at a concentration of 20.0 mg/ml and injected intraperitoneally (i.p.) into mice at a volume of 20 ml/kg body weight. For i.p. administration, dipyridamole was dissolved in PBS at a concentration of 3.3 mg/ml and injected at a volume of 12 ml/kg body weight. [^11^C]Methane was produced in a PETtrace cyclotron equipped with a methane target system (GE Healthcare, Uppsala, Sweden). The unlabeled reference standards 6-bromo-7-methylpurine and *S*-(6-(7-methylpurinyl))glutathione were obtained as gifts from Dr. Toshimitsu Okamura (National Institute of Radiological Sciences, Chiba, Japan).

### Radiotracer Synthesis

The synthesis of 6-bromo-7-[^11^C]methylpurine was slightly modified compared to the published method [13] and is described in detail in the Electronic Supplementary Material. 6-Bromo-7-[^11^C]methylpurine was formulated in 0.9 % (*w*/*v*) aqueous saline for i.v. injection into animals. Radiochemical purity of 6-bromo-7-[^11^C]methylpurine was greater than 98 % and molar activity at the end of synthesis was 444 ± 203 GBq/μmol.

### Animals

Female wild-type, homozygous *Abcc1*^(−/−)^ and heterozygous *Abcc1*^(+/−)^ mice with a C57BL/6J genetic background were obtained from the University of Oslo (Oslo, Norway). Female *Abcc4*^(−/−)^ mice with a C57BL/6 genetic background were obtained from the St. Jude Children’s Research Hospital (Memphis, TN, USA). At the time of experiment, animals weighed 25 ± 4 g. Animals were used in the experiments after being kept for at least one week in our animal facility. Approval was obtained from the competent authority (Amt der Niederösterreichischen Landesregierung) for conducting this study.

### Experimental Design

An overview of animal groups examined in this study is given in Table 1. Groups of wild-type, *Abcc4*^(−/−)^, *Abcc1*^(−/−)^, and *Abcc1*^(+/−)^ mice underwent a baseline PET scan with 6-bromo-7-[^11^C]methylpurine at 30 min after i.p. injection of vehicle solution (PBS). Further groups of wild-type and *Abcc4*^(−/−)^ mice were scanned with 6-bromo-7-[^11^C]methylpurine at 30 min after i.p. injection of the MRP inhibitor MK571 (300 mg/kg). The dosage of MK571 was selected based on previous work by Okamura et al. [12]. One group of wild-type mice underwent a 6-bromo-7-[^11^C]methylpurine PET scan at 30 min after i.p. injection of the nucleoside transporter inhibitor dipyridamole (40 mg/kg). The dosage of dipyridamole was selected based on maximum solubility. In addition, groups of wild-type and *Abcc1*^(−/−)^ mice were used to determine the percentage of unchanged 6-bromo-7-[^11^C]methylpurine in different tissues and fluids.

**Table 1.** Overview of examined animal groups and numbers

	Baseline	MK571 (300 mg/kg) ^a^	Dipyridamole (40 mg/kg)^a^	Metabolism^b^
Wild-type	6	7	2	3
*Abcc4* ^(−/−)^	4	4	–	–
*Abcc1* ^(−/−)^	6	–	–	3
*Abcc1* ^(+/−)^	2	–	–	–

^a^Injected intraperitoneally at 30 min before injection of 6-bromo-7-[^11^C]methylpurine

^b^No PET imaging performed

### PET Imaging

During all experimental procedures, animals were kept under isoflurane anesthesia. PET imaging was conducted on a microPET Focus220 scanner (Siemens Medical Solutions, Knoxville, TN, USA). Mice were i.p. injected under anesthesia at 30 min before start of the PET scan either with vehicle solution (PBS), or with MK571 (300 mg/kg) or with dipyridamole (40 mg/kg). Subsequently, 6-bromo-7-[^11^C]methylpurine (29 ± 9 MBq in a volume of 0.1 ml, 0.17 ± 0.11 nmol) was administered as an i.v. bolus *via* the lateral tail vein, and a 90-min dynamic PET scan (energy window: 250–750 keV; timing window: 6 ns) was initiated at the start of radiotracer injection. At the end of the PET scan, a blood sample was collected from the retro-bulbar plexus and animals were euthanized. Blood was centrifuged to obtain plasma and aliquots of blood and plasma were counted for radioactivity in a gamma counter (Wizard 1470; Perkin-Elmer, Waltham, MA, USA).

### PET Data Analysis

The PET data were sorted into 25 frames with a duration increasing from 5 s to 20 min. Fourier re-binning of the three-dimensional sinograms followed by a two-dimensional filtered back-projection with a ramp filter resulting in a voxel size of 0.4 × 0.4 × 0.796 mm^3^ was used to reconstruct the PET data. Using AMIDE software [15], brain, right lung, left kidney, urinary bladder, liver, duodenum, intestine, and gall bladder were manually outlined on the PET images to derive concentration-time curves expressed in units of standardized uptake value (SUV = (radioactivity per g/injected radioactivity) × body weight). In addition, the left ventricle of the heart was outlined to obtain an image-derived blood curve. For all organs, the area under the concentration-time curve (AUC) from 0 to 90 min after radiotracer injection was calculated using Prism 7 software (GraphPad Software, La Jolla, CA, USA) and organ-to-blood AUC ratios were calculated. From the log-transformed concentration-time curves in blood, brain, lung, kidney, and liver, the elimination rate constant (*k*_elimination_) of radioactivity was determined by linear regression analysis from 17.5 to 80 min after radiotracer injection. A graphical analysis method (integration plot) [16] was used to estimate the rate constant for transfer of radioactivity from blood into different organs (brain, kidney, and liver, *k*_uptake_, ml/min/g tissue) using data measured from 0.3 to 3.5 min after radiotracer injection, as described in detail before [17]. Moreover, the rate constant for tubular secretion of radioactivity (*k*_urine_, min^−1^) was determined from 12.5 to 65 min after radiotracer injection using integration plot analysis [17].

### Assessment of Glutathione Conjugation

In separate groups of wild-type and *Abcc1*^*(−/−)*^ mice, the percentage of unchanged 6-bromo-7-[^11^C]methylpurine and its glutathione conjugate *S*-(6-(7-[^11^C]methylpurinyl))glutathione in different organs and fluids was analyzed by radio-thin layer chromatography (TLC) as described in the Electronic Supplementary Material.

### Analysis of MK571 Concentrations in Plasma and Kidneys

MK571 concentrations in plasma and kidneys were determined as described in the Electronic Supplementary Material.

### Statistical Analysis

Differences between two groups were analyzed with a two-sided *t* test and between multiple groups by one-way ANOVA followed by a Tukey’s multiple comparison test using Prism 7 software. The level of statistical significance was set to a *p* value of less than 0.05. All values are given as mean ± standard deviation (SD) unless stated otherwise.

## Results

### Assessment of Glutathione Conjugation

We used radio-TLC to determine the percentage of unchanged 6-bromo-7-[^11^C]methylpurine and its glutathione conjugate *S*-(6-(7-[^11^C]methylpurinyl))glutathione in different tissues and fluids of wild-type and *Abcc1*^(−/−)^ mice at 15 min after radiotracer injection (Table 2). In none of the investigated tissues and fluids unchanged 6-bromo-7-[^11^C]methylpurine could be detected. In urine and bile, the majority of radioactivity was composed of the radiolabeled glutathione conjugate, while in plasma, brain, kidney, and liver approximately one-half of radioactivity was composed of the glutathione conjugate and the other half of unidentified radiolabeled species, which were not identical with 6-bromo-7-[^11^C]methylpurine. In urine and plasma, the percentage of *S*-(6-(7-[^11^C]methylpurinyl))glutathione was significantly lower in *Abcc1*^(−/−)^ mice than in wild-type mice.

**Table 2.** Percentage of *S*-(6-(7-[^11^C]methylpurinyl))glutathione in different mouse tissues and fluids collected at 15 min after i.v. injection of 6-bromo-7-[^11^C]methylpurine

	Wild-type	*Abcc1* ^(−/−)^
*n*	3	3
Plasma	51 ± 2	43 ± 2^a^
Brain	60 ± 8	52 ± 0.4
Liver	37 ± 2	32 ± 4
Kidney	58 ± 2	63 ± 1
Bile	81 ± 5	79 ± 1
Urine	77 ± 4	63 ± 3^a^

^a^Significantly different compared to wild-type (two-sided *t* test)

### Influence of MK571 on Whole Body Distribution and Excretion of 6-Bromo-7-[^11^C]Methylpurine-Derived Radioactivity

**Fig. 1:**
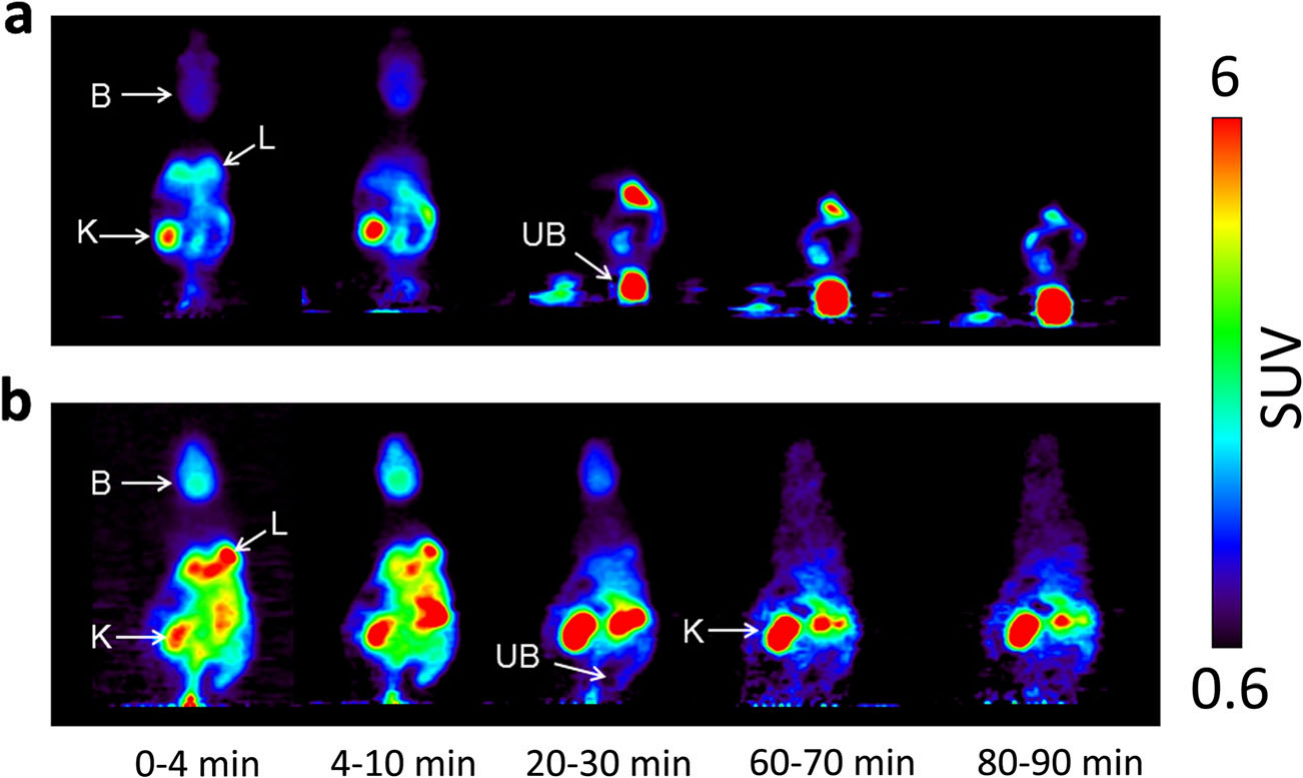
Representative serial coronal whole-body PET images obtained after i.v. injection of 6-bromo-7-[^11^C]methylpurine into **a** a wild-type mouse pre-treated with vehicle or **b** with MK571 (300 mg/kg, i.p.) at 30 min before the PET scan. Radiation scale is given as standardized uptake value (SUV). Anatomic structures are labeled by arrows: *K* left kidney, *UB* urinary bladder, *B* brain, *L* right lung.

**Fig. 2:**
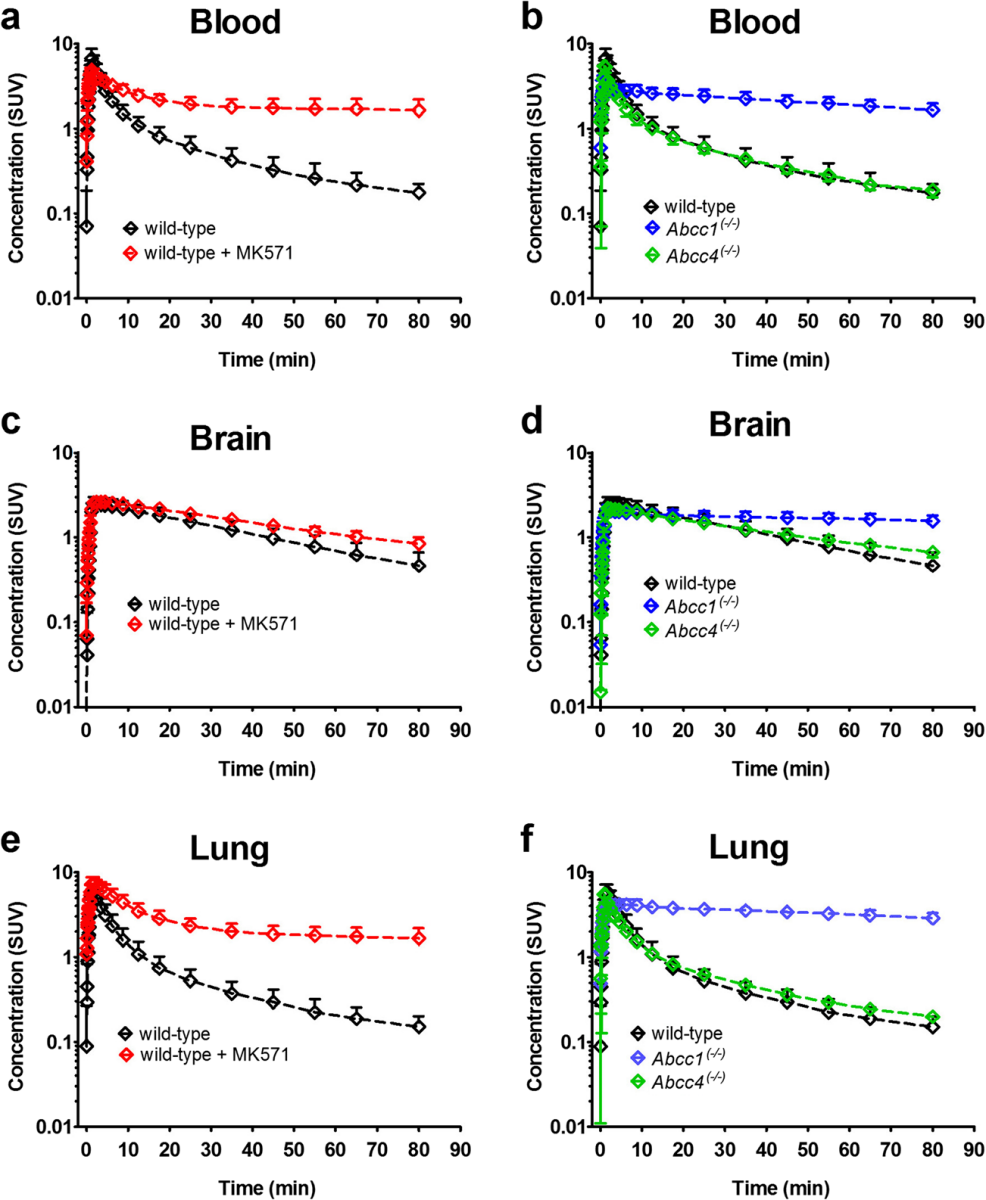
Concentration-time curves (mean standardized uptake value, SUV ± SD) in **a** the blood (derived from the left ventricle of the heart), **c** the whole brain, and **e** the right lung of wild-type mice pre-treated at 30 min before PET either with vehicle or with MK571 (300 mg/kg, i.p.). In **b**, **d**, **f**, concentration-time curves (mean standardized uptake value, SUV ± SD) in the blood, the whole brain, and the right lung are shown for wild-type, *Abcc1*^(−/−)^ and *Abcc4*^(−/−)^ mice.

**Fig. 3:**
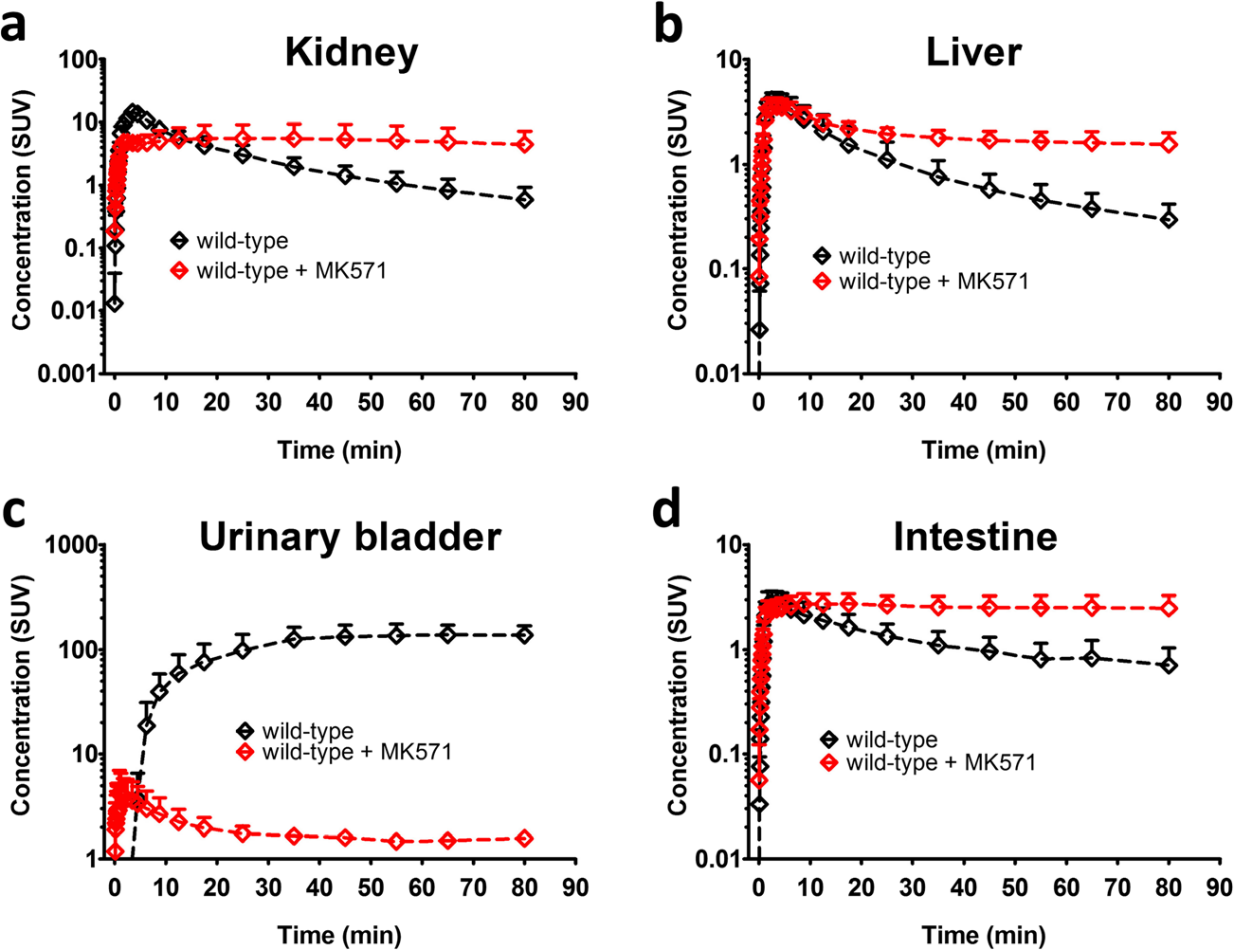
Concentration-time curves (mean standardized uptake value, SUV ± SD) in **a** the left kidney, **b** the liver, **c** the urinary bladder, and **d** the intestine (including the gall bladder and the duodenum) of wild-type mice pre-treated at 30 min before PET either with vehicle or with MK571 (300 mg/kg, i.p.).

To assess the influence of MRPs on tissue distribution and excretion of 6-bromo-7-[^11^C]methylpurine-derived radioactivity, we performed PET scans in wild-type mice, which were pre-treated before the scan either with vehicle or with MK571. Whole-body PET images depicting the distribution of 6-bromo-7-[^11^C]methylpurine-derived radioactivity over time in one vehicle-treated and one MK571-treated wild-type mouse are shown in Fig. 1. To obtain image-derived blood concentration-time curves, a region of interest was drawn around the left ventricle of the heart. A good correlation was found between the image-derived blood radioactivity concentrations and radioactivity measured in venous blood at the end of the PET scan (Pearson correlation coefficient *r* = 0.980, *p* < 0.0001, slope = 0.77 ± 0.04). Following pre-treatment with MK571, blood radioactivity concentrations were significantly increased and elimination of radioactivity from the blood was markedly delayed (Fig. 2a). In MK571-pre-treated animals, the AUC of the image-derived blood concentration-time curves was increased by 2.8-fold and *k*_elimination,blood_ was decreased by 4.9-fold, as compared with vehicle-treated animals. MK571 pre-treatment caused in parallel to changes in blood pharmacokinetics pronounced changes in the shape of the concentration-time curves in all investigated organs (Figs. 2 and 3). In brain, lung, kidney, and liver, *k*_elimination_ values were significantly decreased by 1.5-, 3.0-, 8.5-, and 4.3-fold, respectively (Fig. 5). In MK571-treated animals, organ-to-blood AUC ratios were significantly decreased, by 2.2-fold in the brain and by 1.5-fold in the liver, but not in the other investigated organs. We also estimated the rate constants for transfer of radioactivity from blood into the respective organs (*k*_uptake_) with integration plot analysis (Fig. 6). *K*_uptake_ values in vehicle-treated wild-type mice were 0.749 ± 0.228 ml/min/g tissue in the kidneys and 0.211 ± 0.057 ml/min/g tissue in the liver, which corresponded to extraction ratios of 0.18 and 0.21, respectively (assuming renal and hepatic blood flow rates of 4.1 and 1.0 ml/min/g tissue) [18]. The only organ in which *k*_uptake_ was significantly changed after MK571 treatment were the kidneys, showing a decrease by 3.5-fold (Fig. 6a). Over the time course of the PET scan, radioactivity appeared to mainly undergo urinary excretion in vehicle-treated wild-type mice (Fig. 1). We estimated the rate constant for transfer of radioactivity from the kidneys into urine (*k*_urine_) using integration plot analysis (Fig. S1). Pre-treatment with MK571 completely abolished urinary excretion of radioactivity (Fig. 3c) and decreased *k*_urine_ by 42.6-fold (Fig. 5f). We also found uptake of radioactivity in the intestine, but intestinal concentrations of radioactivity were considerably lower than in the urinary bladder. Intestinal AUCs were significantly (2.1-fold) higher in MK571-treated mice compared to vehicle-treated mice. The rate constant for transfer of radioactivity from the liver *via* bile into the intestine (*k*_bile_) could not be estimated as the slopes of the linear parts of the integration plots were negative.

MK571 concentrations at approximately 120 min after i.p. administration of MK571 were 251 ± 58 μmol/L in the plasma and 544 ± 58 μmol/kg in the kidneys of wild-type mice.

### Influence of ABCC1 and ABCC4 on Whole Body Distribution and Excretion of 6-Bromo-7-[^11^C]Methylpurine-Derived Radioactivity

**Fig. 4:**
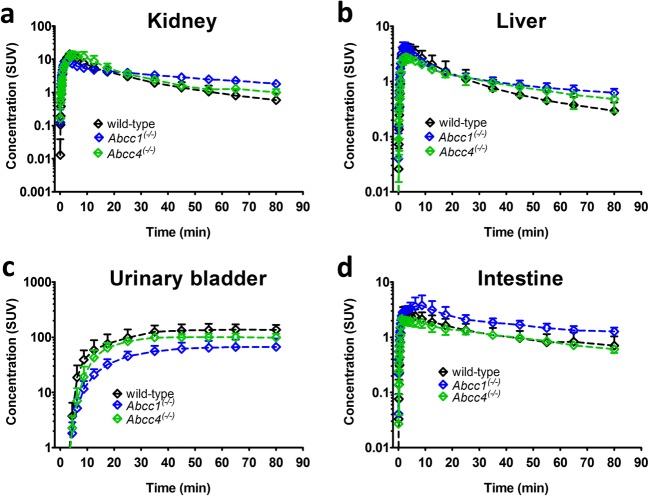
Concentration-time curves (mean standardized uptake value, SUV ± SD) in **a** the left kidney, **b** the liver, **c** the urinary bladder, and **d** the intestine (including the gall bladder and the duodenum) of wild-type, *Abcc1*^(−/−)^, and *Abcc4*^(−/−)^ mice.

**Fig. 5:**
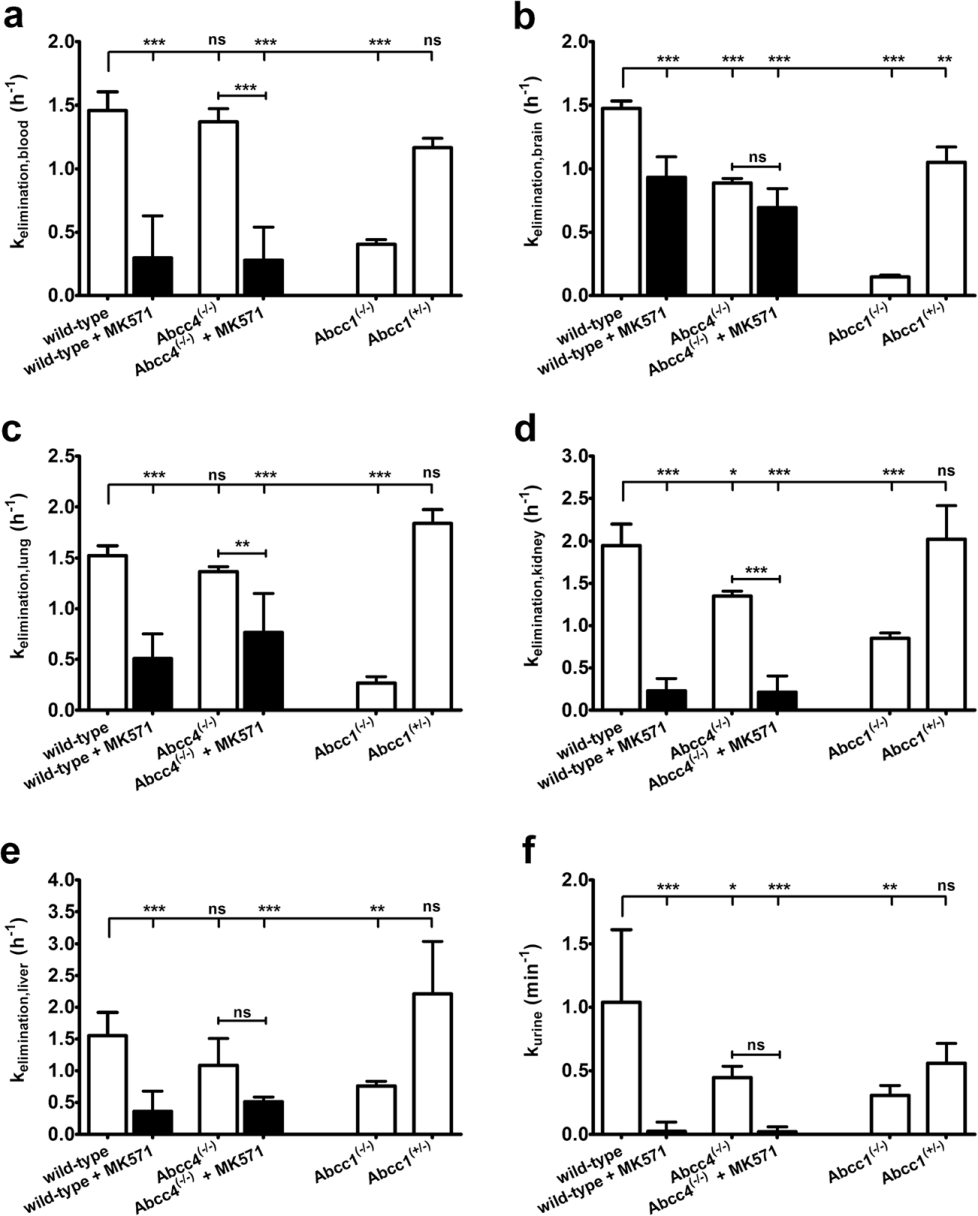
*K*_elimination_ values (mean h^−1^ ± SD) of radioactivity from **a** the blood (derived from the left ventricle of the heart), **b** the whole brain, **c** the right lung, **d** the left kidney, and **e** the liver of wild-type and *Abcc4*^(−/−)^ mice pre-treated at 30 min before PET either with vehicle or with MK571 (300 mg/kg, i.p.), of *Abcc1*^(−/−)^ mice and *Abcc1*^(+/−)^ mice. In **f**, *k*_urine_ (mean min^−1^ ± SD) is shown for the same groups as in **a**–**e** (*ns* not significant, *p < 0.05, **p < 0.01, and ***p < 0.001, for comparison with wild-type mice using one-way ANOVA with Tukey’s multiple comparison test).

**Fig. 6:**
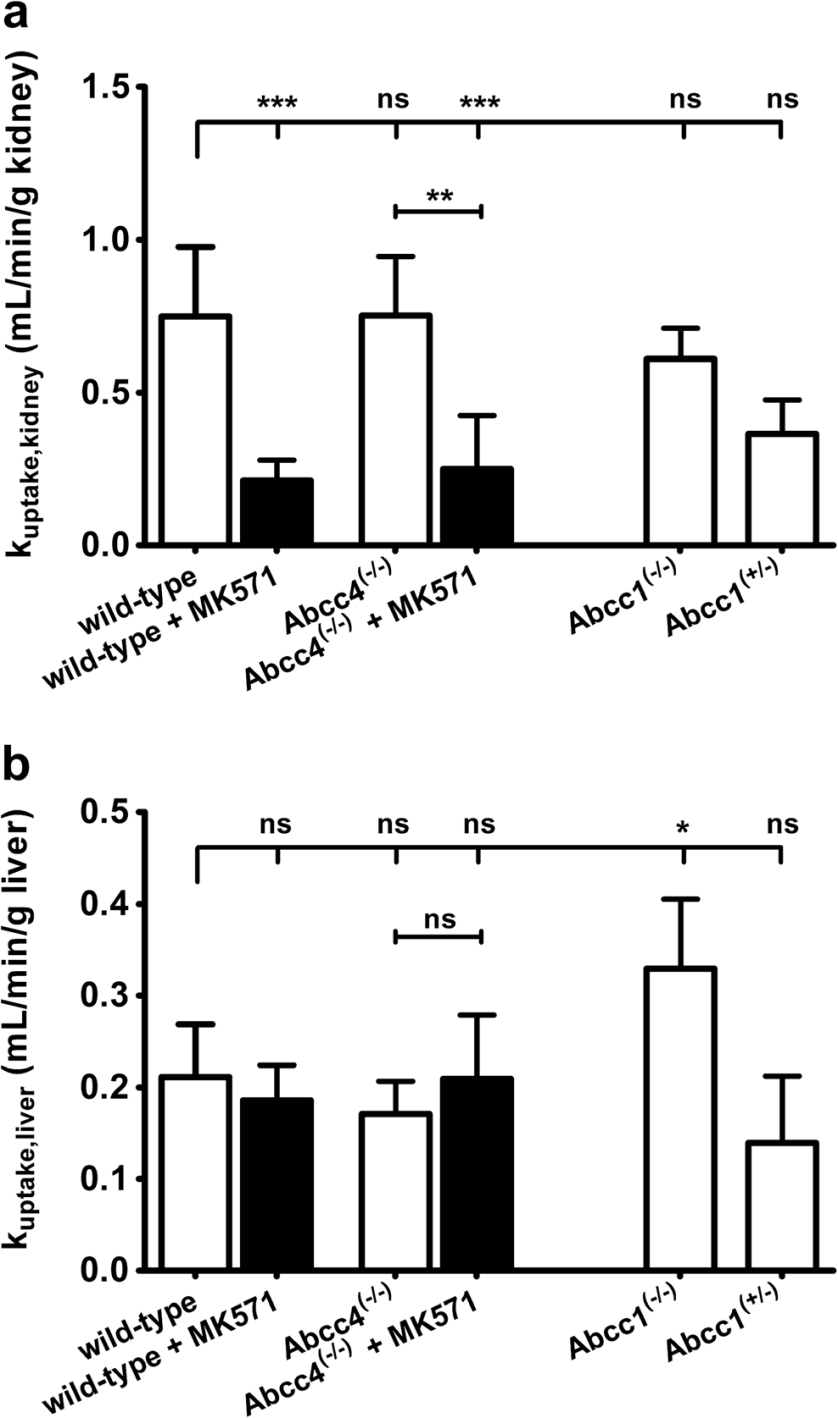
*K*_uptake_ values (mean ml/min/g tissue ± SD) of radioactivity from the blood into **a** the left kidney and **b** the liver of wild-type and *Abcc4*^(−/−)^ mice pre-treated at 30 min before PET either with vehicle or with MK571 (300 mg/kg, i.p.), of *Abcc1*^(−/−)^ mice and *Abcc1*^(+/−)^ mice (*ns* not significant, *p < 0.05, **p < 0.01, and ***p < 0.001, for comparison with wild-type mice using one-way ANOVA with Tukey’s multiple comparison test).

To investigate which MRP subtypes were involved in the tissue distribution and urinary excretion of 6-bromo-7-[^11^C]methylpurine-derived radioactivity, we examined *Abcc1*^(−/−)^ and *Abcc4*^(−/−)^ mice. Knockout of *Abcc1* caused pronounced changes in blood, brain, and lung concentration-time curves, which were comparable to the changes seen in MK571-treated wild-type mice (Figs. 2 and Fig. 4). Blood AUCs in *Abcc1*^(−/−)^ mice were increased by 3.0-fold as compared with wild-type mice. Plasma-to-blood ratios of radioactivity, determined from the venous blood sample collected at the end of the PET scan, were decreased by 2.4-fold (wild-type: 1.46 ± 0.14, *Abcc1*^(−/−)^: 0.60 ± 0.04). *Abcc1*^(−/−)^ mice showed 3.6-, 9.4-, and 5.7-fold reduced *k*_elimination_ values in the blood, the brain, and the lungs, respectively (Fig. 5). *K*_elimination_ values were also significantly decreased in the kidneys and the liver of *Abcc1*^(−/−)^ mice relative to wild-type mice, but to a lesser degree than in the brain and the lungs. *K*_urine_ values were significantly decreased in *Abcc1*^(−/−)^ mice by 3.4-fold. In the liver of *Abcc1*^(−/−)^ mice, *k*_uptake_ values were significantly increased by 1.6-fold relative to wild-type mice (Fig. 6b).

*Abcc4*^(−/−)^ mice also showed significantly decreased *k*_elimination_ values in some of the investigated organs, *i.e*., brain (1.5-fold) and kidneys (1.4-fold) (Fig. 5). *K*_urine_ values of *Abcc4*^(−/−)^ mice were significantly lower (2.3-fold) than in wild-type mice. We also examined a group of *Abcc4*^(−/−)^ mice which was pre-treated with MK571. The effect of MK571 pre-treatment in different organs of *Abcc4*^(−/−)^ mice was comparable to the effect seen in wild-type mice (Figs. 5 and Fig. 6).

To assess the sensitivity of 6-bromo-7-[^11^C]methylpurine PET to measure moderate changes in ABCC1 expression, we examined heterozygous *Abcc1* knockout mice (*Abcc1*^(+/−)^), which are expected to have a 50 % reduction in ABCC1 expression levels in different tissues. Heterozygous *Abcc1*^(+/−*)*^ mice showed, relative to wild-type mice, a much smaller but significant reduction in *k*_elimination_ values from the brain as compared with homozygous *Abcc1*^(−/−)^ mice (1.3-fold *versus* 9.4-fold reduction in *k*_elimination,brain_ relative to wild-type mice), but not in the other investigated organs (Fig. 5).

To determine whether nucleoside transporters play a role in the distribution of 6-bromo-7-[^11^C]methylpurine-derived radioactivity from blood into brain, we examined wild-type mice pre-treated with the nucleoside transporter inhibitor dipyridamole. Brain concentration-time curves were similar in vehicle- and dipyridamole-treated wild-type mice (Fig. S2) and *k*_uptake,brain_ values were not significantly different between the two groups (*k*_uptake,brain_ vehicle: 0.113 ± 0.028 ml/min/g brain, dipyridamole: 0.120 ± 0.004 ml/min/g brain).

## Discussion

6-Bromo-7-[^11^C]methylpurine has been developed as a PET tracer to assess ABCC1 activity in the brain and in the lungs [12, 13]. Since MRPs are important transport proteins in the kidneys and the liver, we were interested in finding out if 6-bromo-7-[^11^C]methylpurine can be used to measure the activities of MRPs in these organs. To this end, we performed PET scans in C57BL/6J wild-type mice pre-treated with vehicle or the non-subtype selective MRP inhibitor MK571 [19–21].

Radio-TLC analysis showed that the majority of radioactivity in most investigated organs and fluids was composed of *S*-(6-(7-[^11^C]methylpurinyl))glutathione, while no 6-bromo-7-[^11^C]methylpurine could be detected (Table 2). This is consistent with glutathione-*S*-transferases being expressed in most tissues, including brain, lung, kidneys, and liver [22]. Our findings are in good agreement with the results obtained by Okamura et al. in FVB mice [12, 13]. However, we also observed other, unidentified radiolabeled species, which may be degradation products of the glutathione conjugate generated by metabolic enzymes, such as γ-glutamyl transpeptidase [12]. The presence of at least two different radiolabeled species in different proportions in different tissues and fluids (Table 2) constitutes a disadvantage of 6-bromo-7-[^11^C]methylpurine for accurate quantification of transporter function in mice.

MK571 treatment dramatically changed the blood pharmacokinetics of radioactivity by increasing the AUC and by decreasing *k*_elimination,blood_ (Fig. 2). This pointed to changes in the disposition of radioactivity in clearance organs, which may have been caused by inhibition of MRPs by MK571. Our PET data showed that 6-bromo-7-[^11^C]methylpurine-derived radioactivity mainly underwent urinary excretion (Fig. 1). MK571 treatment decreased *k*_uptake,kidney_, delayed washout of radioactivity from the kidney (*k*_elimination,kidney_), and reduced *k*_urine_ almost to zero. While the reduction in *k*_uptake,kidney_ may have been caused by inhibition of kidney uptake transporter(s) by MK571, the pronounced decreases in *k*_elimination,kidney_ and *k*_urine_ suggest that MRP activity is a rate-limiting step in the urinary excretion of 6-bromo-7-[^11^C]methylpurine-derived radioactivity. The candidate MRP subtypes are Abcc2 and Abcc4, which are both expressed in the brush border membrane of kidney proximal tubule cells, where they promote excretion of drugs and drug conjugates into urine [23]. The mean total MK571 concentration in the kidneys was 544 μM. Reported apparent tissue unbound fractions in rats were 0.33 for the (*S*)-enantiomer and 0.18 for (*R*)-enantiomer of MK571 [24]. This would correspond to kidney unbound concentrations of MK571 in mice of 180 and 98 μM, respectively, which were several times above the *in vitro* half-maximum inhibitory concentrations (IC_50_) of MK571 for inhibition of substrate transport by ABCC2 and ABCC4 (IC_50_: ABCC2: 4 μmol/L, ABCC4: 10 μmol/L) [20, 21]. MK571 was reported to undergo glutathione conjugation in rats [25]. Therefore, it cannot be excluded that competition of MK571 with 6-bromo-7-[^11^C]methylpurine for glutathione conjugation may have contributed to the MRP-inhibitory effects of MK571 in the mouse kidney in causing a reduction in renal excretion of radioactivity.

To elucidate which MRPs were involved in urinary excretion of radioactivity, we studied *Abcc4*^(−/−)^ mice. *Abcc4*^(−/−)^ mice showed significantly decreased *k*_elimination,kidney_ and *k*_urine_ values, indicating that Abcc4 contributed to renal excretion of 6-bromo-7-[^11^C]methylpurine-derived radioactivity. However, as compared to treatment with MK571, the effect of *Abcc4* knockout on *k*_elimination,kidney_ and *k*_urine_ values was smaller, suggesting that other MRP subtype(s) also contributed to renal excretion of radioactivity. This other transporter may have been Abcc2, which shares a very similar substrate specificity with Abcc1. However, we were not able to examine the role of Abcc2 as we had no access to *Abcc2*^(−/−)^ mice. Interestingly, *Abcc1* knockout caused more pronounced decreases in *k*_elimination,kidney_ and *k*_urine_ values than *Abcc4* knockout (Fig. 5d, f). This is surprising, as Abcc1 is not expressed in the apical membrane and only very weakly expressed in the basolateral membrane of kidney proximal tubule cells [26]. Based on the localization of Abcc1 in the mouse kidneys, *Abcc1* knockout would have been expected to lead to an increase in *k*_uptake,kidney_, which was not observed in our study. Interestingly, radio-TLC analysis revealed significantly lower percentages of *S*-(6-(7-[^11^C]methylpurinyl))glutathione in the urine and plasma of *Abcc1*^(−/−)^ mice as compared with wild-type mice (Table 2). This points to differences in glutathione conjugation of 6-bromo-7-[^11^C]methylpurine between *Abcc1*^(−/−)^ and wild-type mice, which may have contributed to the observed decrease in renal excretion of 6-bromo-7-[^11^C]methylpurine-derived radioactivity in *Abcc1*^(−/−)^ mice.

Next to the kidneys, we also observed radioactivity distribution to the liver and the intestine. However, the rate constant for transfer of radioactivity from the liver into the intestine *via* bile could not be estimated with integration plot analysis, indicating that hepatobiliary excretion of radioactivity over the time course of the PET scan was negligible. Distribution of radioactivity to the intestine may have been caused by direct secretion of radioactivity from the blood into the intestine. In the mouse liver, mRNA and protein levels of Abcc1 are very low, but may be induced by exposure to xenobiotics or by bile duct ligation [27, 28]. In our study, *Abcc1* knockout led to a significant increase in *k*_uptake,liver_ (Fig. 6b), which may point to a basolateral localization of low Abcc1 expression levels in mouse hepatocytes. *Abcc1* knockout mice had significantly decreased plasma-to-blood ratios of radioactivity as compared to wild-type mice indicating increased retention of 6-bromo-7-[^11^C]methylpurine-derived radioactivity in cellular blood components expressing Abcc1, such as mononuclear cells [29].

Our data confirm previous results that 6-bromo-7-[^11^C]methylpurine can measure ABCC1 activity in the brain and in the lungs [12, 13]. In the brain, ABCC1 is highly expressed in the basolateral membrane of epithelial cells of the choroid plexus, where it promotes transport of its substrates from the epithelial cells into blood thereby contributing to the elimination of anionic substances from the brain *via* the blood-cerebrospinal fluid barrier (BCSFB) [30]. In addition, ABCC1 is expressed in glial cells (astrocytes) [31]. However, the expression of ABCC1 in the luminal membrane of brain capillary endothelial cells forming the blood-brain barrier (BBB) remains controversial [32, 33]. In agreement with the results by Okamura et al. [13], we observed a pronounced reduction in *k*_elimination_ of radioactivity from the brain in *Abcc1*^(−/−)^ relative to wild-type mice. Similarly, MK571 pre-treatment led to a reduction in *k*_elimination_ of radioactivity from the brain, which indicates that MK571, which has so far been mostly used in *in vitro* experiments, can serve as an *in vivo* inhibitor of Abcc1. As discussed before [13], brain disposition of 6-bromo-7-[^11^C]methylpurine involves several steps: (i) distribution of 6-bromo-7-[^11^C]methylpurine across the BBB into brain parenchyma, (ii) intracellular conversion of 6-bromo-7-[^11^C]methylpurine into its glutathione conjugate (*e.g*., in astrocytes), (iii) efflux of the glutathione conjugate into brain interstitium, and (iv) elimination of the glutathione conjugate from the brain. It is currently not known, which of these steps is the rate-limiting step in radioactivity elimination from the brain, *i.e*., *Abcc1* knockout may have either impeded efflux of the glutathione conjugate from astrocytes and thereby reduced *k*_elimination,brain_ or it could have also inhibited elimination of radioactivity across the BCSFB or the BBB.

As 6-bromo-7-[^11^C]methylpurine is a nucleobase analogue, its brain uptake may be mediated by nucleoside transporters at the BBB, *i.e*., equilibrative nucleoside transporter (ENT) 1 and 2 (SLC29A1 and SLC29A2), which recognize nucleobases as their substrates [34]. To investigate a possible role of Slc29a1 and Slc29a2 in brain uptake of 6-bromo-7-[^11^C]methylpurine, we examined a small group of wild-type mice pre-treated with the prototypical nucleoside transporter inhibitor dipyridamole [35], which has been used before *in vivo* in mice to inhibit ENTs [36]. We found no effect of dipyridamole on *k*_uptake,brain_, which suggested that brain uptake of 6-bromo-7-[^11^C]methylpurine was not mediated by these transporters. Dipyridamole was also shown to inhibit *in vitro* ABC transporters including ABCB1 and MRPs [20, 37], but *k*_elimination,brain_ of 6-bromo-7-[^11^C]methylpurine-derived radioactivity was similar in vehicle- and dipyridamole-treated mice (*k*_elimination,brain_ vehicle: 1.37 ± 0.27 h^−1^, dipyridamole: 1.59 ± 0.07 h^−1^).

Previous data with PET tracers for the imaging of ABCB1 activity at the BBB (*i.e*., (*R*)-[^11^C]verapamil and [^11^C]*N*-desmethyl-loperamide) have shown that moderate reductions (< 50 %) in Abcb1a expression at the BBB lead to only very small changes in brain distribution of these radiotracers, as compared to complete *Abcb1a* knockout [38]. This has been attributed to ABCB1 being a high-capacity transporter which can functionally compensate moderate expression changes, so that brain distribution of ABCB1 substrate drugs remains largely unchanged. To assess the sensitivity of 6-bromo-7-[^11^C]methylpurine to measure Abcc1 activity in the brain, we examined heterozygous *Abcc1* knockout (*Abcc1*^(+/−)^) mice, which are expected to have a 50 % reduction in Abcc1 expression [39, 40]. In comparison to homozygous *Abcc1*^(−/−)^ mice, heterozygous *Abcc1*^(+/−)^ mice had only a moderate reduction in *k*_elimination,brain_, which indicates that 6-bromo-7-[^11^C]methylpurine possesses a limited sensitivity to measure moderate changes in ABCC1 expression/function in the brain. In contrast to Abcc1, quantitative targeted absolute proteomics data revealed that Abcc4 is expressed in the luminal membrane of the mouse BBB [33]. Consistent with this, we showed a reduction in *k*_elimination,brain_ in *Abcc4*^(−/−)^ relative to wild-type mice, which indicated that Abcc4 contributed to elimination of 6-bromo-7-[^11^C]methylpurine-derived radioactivity from the brain. It should be noted, however, that species differences have been reported between mice and humans in the abundance of different MRP subtypes in different tissues. While Abcc4 was the third most abundant ABC transporter at the mouse BBB, ABCC4 was below the limit of quantification at the human BBB [41]. Therefore, ABCC4 would most likely exert a negligible impact on the brain kinetics of 6-bromo-7-[^11^C]methylpurine-derived radioactivity in humans.

In the lungs, ABCC1 is expressed in the basolateral membrane of different pulmonary epithelial cell types (airway epithelial cells, alveolar type 2 and type 1 cells) [42]. ABCC1 may protect lung tissue from xenobiotics by restricting distribution of i.v. administered substances from blood into lung parenchyma and by promoting elimination of inhaled drugs or drug conjugates from the lungs into blood [42]. In accordance with previous findings [12], we observed pronounced decreases in *k*_elimination_ of 6-bromo-7-[^11^C]methylpurine-derived radioactivity from the lungs, both in *Abcc1*^(−/−)^ and in MK571-treated wild-type mice. Of all studied organs, the effects of *Abcc1* knockout or inhibition were highest in the lungs, which was consistent with ABCC1 being the most abundantly expressed ABC transporter in the lungs [43]. It should be noted, however, that PET measures total radioactivity concentration in tissue including the vascular space. As some of the investigated organs (kidney, liver, and lungs) contain a large blood pool, it cannot be excluded that the observed reductions in tissue *k*_elimination_ values may be partly due to changes in *k*_elimination,blood_ values. Absence of changes in pulmonary disposition of radioactivity in *Abcc4*^(−/−)^ mice was consistent with low expression levels of this transporter in the lungs [43].

## Conclusion

We assessed the utility of 6-bromo-7-[^11^C]methylpurine to measure the activity of MRPs in excretory organs by performing PET scans in wild-type, *Abcc4*^(−/−)^, and *Abcc1*^(−/−)^ mice, with and without pre-treatment with the prototypical MRP inhibitor MK571. Our data revealed pronounced effects of MK571 treatment on blood, kidney, and urinary bladder concentration time-curves of 6-bromo-7-[^11^C]methylpurine-derived radioactivity. Pharmacokinetic analysis indicated that MK571 completely abolished urinary excretion of radioactivity suggesting that MRP activity is a rate-limiting step in the renal clearance of 6-bromo-7-[^11^C]methylpurine-derived radioactivity. Experiments in *Abcc4*^(−/−)^ mice revealed that Abcc4 contributed to the urinary excretion of 6-bromo-7-[^11^C]methylpurine-derived radioactivity. Our data suggest that 6-bromo-7-[^11^C]methylpurine may be of use to assess the activity of MRPs in the kidneys, for which no PET tracers have been described yet. However, further efforts are needed to identify the MRP subtypes involved in the renal excretion of 6-bromo-7-[^11^C]methylpurine-derived radioactivity. Moreover, our data confirm and extend previous findings that 6-bromo-7-[^11^C]methylpurine can measure the activity of Abcc1 in the brain and in the lungs.

## Electronic supplementary material

ESM 1 (PDF 463 kb)
