# Supplementary material for: Influence of Multidrug Resistance-Associated Proteins on the Excretion of the ABCC1 Imaging Probe 6-Bromo-7-[11C]Methylpurine in Mice
Source: Mol Imaging Biol. 2018 Jun 25;21(2):306–16. doi: 10.1007/s11307-018-1230-y (PMC6449286; doi:10.1007/s11307-018-1230-y)
Supplement: Supplementary file 1 — (PDF 463 kb) [file 11307_2018_1230_MOESM1_ESM.pdf]

Electronic Supplementary Material

Influence of Multidrug Resistance-Associated Proteins on the Excretion of the ABCC1 Imaging Probe 6-Bromo-7-[^{11}C]Methylpurine in Mice

Journal: Molecular Imaging and Biology

Viktoria Zoufal,[1] Severin Mairinger,[1] Markus Krohn,[2] Thomas Wanek,[1] Thomas Filip,[1] Michael Sauberer,[1] Johann Stanek,[1,3] Alexander Traxl,[1] John D. Schuetz,[4] Claudia Kuntner,[1] Jens Pahnke,[2,5,6] Oliver Langer,[1,3,7]

[1] *Biomedical Systems, Center for Health & Bioresources, AIT Austrian Institute of Technology GmbH, Seibersdorf, Austria*

[2] *Department of Neuro-/Pathology, University of Oslo (UiO) and Oslo University Hospital (OUS), Oslo, Norway*

[3] *Department of Clinical Pharmacology, Medical University of Vienna, Vienna, Austria*

[4] *Department of Pharmaceutical Sciences, St. Jude Children's Research Hospital, Memphis, Tennessee, USA*

[5] *LIED, University of Lübeck, Germany*

[6] *Leibniz-Institute of Plant Biochemistry, Halle, Germany*

[7] *Division of Nuclear Medicine, Department of Biomedical Imaging and Image-guided Therapy, Medical University of Vienna, Vienna, Austria*

Correspondence to: Oliver Langer; *e-mail*: oliver.langer@ait.ac.at

Radiosynthesis of 6-Bromo-7-[^{11}C]Methylpurine

6-Bromo-7-[^{11}C]methylpurine was synthesized by reacting 6-bromopurine with [^{11}C]methyl triflate. 6-Bromopurine (1 mg, 5.0 µmol) and potassium carbonate (2 mg, 14.5 µmol, 2.9 equivalents) were suspended in DMSO (100 µl) and the supernatant was transferred into the reactor of a Tracerlab FX C Pro synthesis module (GE Healthcare Uppsala, Sweden). [^{11}C]Methyl triflate was synthesized from [^{11}C]methane *via* [^{11}C]methyl iodide according to standard procedures [1] and subsequently transferred into the 6-bromopurine solution. The reaction mixture was heated for 4 min at 80°C. After cooling (35°C) and dilution with 0.9 ml of ethyl acetate, the mixture was injected into a high-performance liquid chromatography (HPLC) system. A Spherisorb 250 x 20 mm HPLC NP column was eluted with ethyl acetate/ethanol (81/19, v/v) at a flow rate of 4 ml/min. The HPLC eluate was monitored in series for radioactivity and ultraviolet (UV) absorption at a wavelength of 254 nm. Using this system, 6-bromopurine and product 6-bromo-7-[^{11}C]methylpurine eluted with retention times of 9.6 - 11 min and 12.4 -13.2 min, respectively. The product fraction was diluted with ethanol (1 ml) and transferred into a 10-ml glass vial. The solvent was removed under vacuum and the product was formulated in 0.9% (w/v) aqueous saline at an approximate concentration of 370 MBq/ml for i.v. injection into animals. Radiochemical purity and molar activity of 6-bromo-7-[^{11}C]methylpurine were determined by analytical radio-HPLC using a Chromolith Performance RP-18e column (5 µM, 100-4.6 mm, Merck KGaA, Darmstadt, Germany) eluted with 5% acetonitrile in aqueous ammonium acetate buffer (0.2 M, pH 5.0) at a flow rate of 1 ml/min. UV detection was performed at a wavelength of 270 nm. The retention time of 6-bromo-7-[^{11}C]methylpurine was 9.2 min on this HPLC system. 6-Bromo-7-[^{11}C]methylpurine was synthesized in a decay-corrected radiochemical yield of 4.3 ± 1.0%, based on [^{11}C]methane, in a total synthesis time of approximately 30 min. Radiochemical purity of 6-bromo-7-[^{11}C]methylpurine was greater than 98% and molar activity at the end of synthesis was 444 ± 203 GBq/µmol.

Assessment of Glutathione Conjugation

6-Bromo-7-[^{11}C]methylpurine (26 ± 8 MBq in a volume of 0.1 ml, 0.15 ± 0.06 nmol) was administered i.v. to wild-type and *Abcc1*$^{(-/-)}$ mice (*n* = 3 per group) under isoflurane anesthesia. After a period of 15 min, blood was collected from the retro-bulbar plexus and animals were euthanized. Brain, liver, kidneys, urine and bile were collected. Blood was centrifuged to obtain plasma and proteins were precipitated by the addition of acetonitrile (1 µl per µl plasma). Liver, kidneys and brain were homogenized using an Ultra Turrax T10 (IKA Laboratory Equipment, Staufen, Germany) and proteins were precipitated with acetonitrile (1 ml per liver, 0.2 ml per kidneys/brain). Urine and bile were diluted with acetonitrile (1 µl per µl urine and bile). All solutions were vortexed and then centrifuged (12,000 x g, 1 min, 21°C). Each supernatant (plasma, brain, liver, kidney, urine, bile, 5 µl each) and diluted radiotracer solution as reference were spotted on silica gel 60F 254 nm TLC plates (10 x 20 cm; Merck, Darmstadt, Germany) and plates were developed in water/acetonitrile/acetic acid (92/8/1, v/v/v). Detection was performed by placing the TLC plates on multisensitive phosphor screens (Perkin-Elmer). The screens were scanned at 300 dpi resolution using a Perkin-Elmer Cyclone® Plus Phosphor Imager. The retardation factor (R_f) of 6-bromo-7-[^{11}C]methylpurine was 0.2, while the radiolabeled glutathione conjugate had an R_f of 0.7 as assessed by use of unlabeled reference standards.

Analysis of MK571 Concentrations in Plasma and Kidneys

In three MK571 pre-treated wild-type animals, plasma and kidneys were collected at the end of the PET scan and analyzed with HPLC for MK571 concentrations using an Agilent 1260 system (Agilent Technologies, Santa Clara, CA, USA) equipped with an Agilent 1260 fluorescence detector (G1321 FLD). Briefly, after adding 250 µl of acetonitrile to 100 µl of plasma, the samples were vortexed and centrifuged (10,000 x g for 3 min at 4°C) and 200 µl of the clear supernatant was diluted with 100 µl of 0.1% aqueous acetic acid. For the sample preparation of the kidneys, 50 µl of water was added per kidney and the tissue was homogenized. 50 µl of 0.2% aqueous acetic acid and 300 µl of acetonitrile were added to the

homogenate and the samples were vortexed and centrifuged (10,000 x g for 3 min at 4°C) to obtain a clear supernatant. The resulting supernatants were injected into the HPLC column. Analysis of MK571 was carried out at 40°C using a Chromolith Performance RP-18e column (5 µm, 100-4.6 mm) preceded by a Chromolith Performance RP-18 pre-column (5 µm, 10-4.6 mm), which was isocratically eluted with acetonitrile/methanol/0.1% aqueous acetic acid (30/20/50, v/v/v) at a flow rate of 2 ml/min. For fluorescence detection, an excitation wavelength of 250 nm and an emission wavelength of 410 nm were used. MK571 eluted with a retention time of 10.1 min. Linear calibration curves were generated by spiking drug-free mouse plasma or kidney homogenate with MK571 (final concentrations ranging from 50 µg to 1,000 µg/ml; average correlation coefficient: > 0.999).

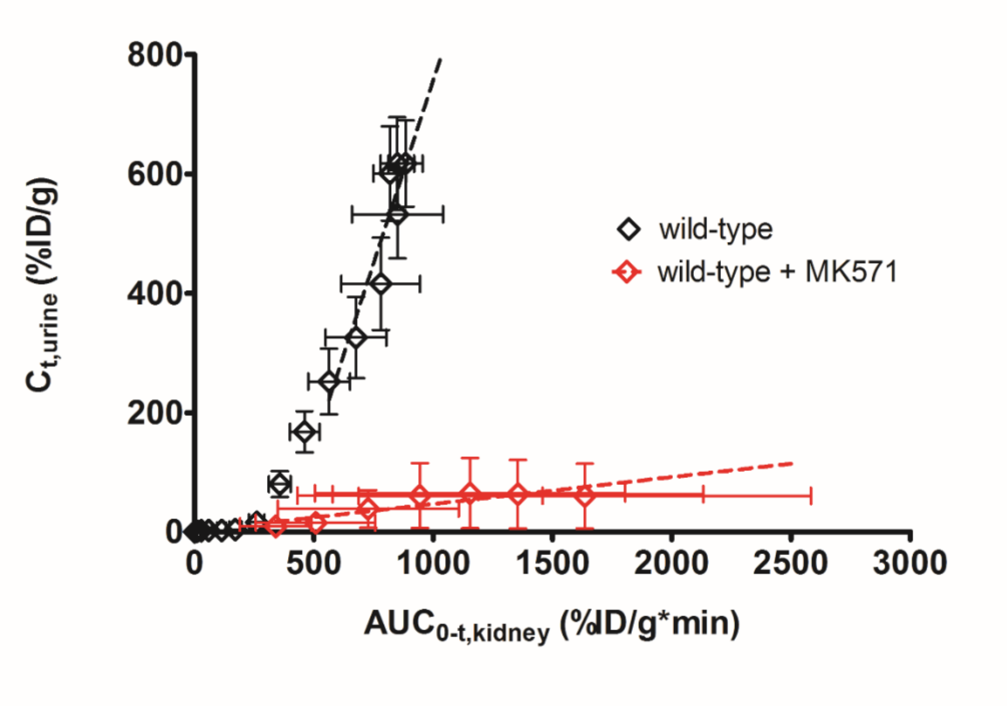

Supplementary Fig 1 Mean (± standard error of the mean) integration plots in wild-type mice pre-treated with vehicle or with MK571 (300 mg/kg, i.p.) at 30 min before the PET scan to determine the rate constant for transfer of radioactivity from the kidneys into urine k_{urine} (min^{-1}). $C_{t,urine}$: radioactivity concentration in the urinary bladder at time t (given in percent injected dose per g tissue, %ID/g); $AUC_{0\text{-}t,kidney}$: area under the concentration-time curve in the left kidney from time 0 to time t. The slope of the linear regression line (from 12.5 to 65 min after radiotracer injection, broken line) corresponds to k_{urine}.

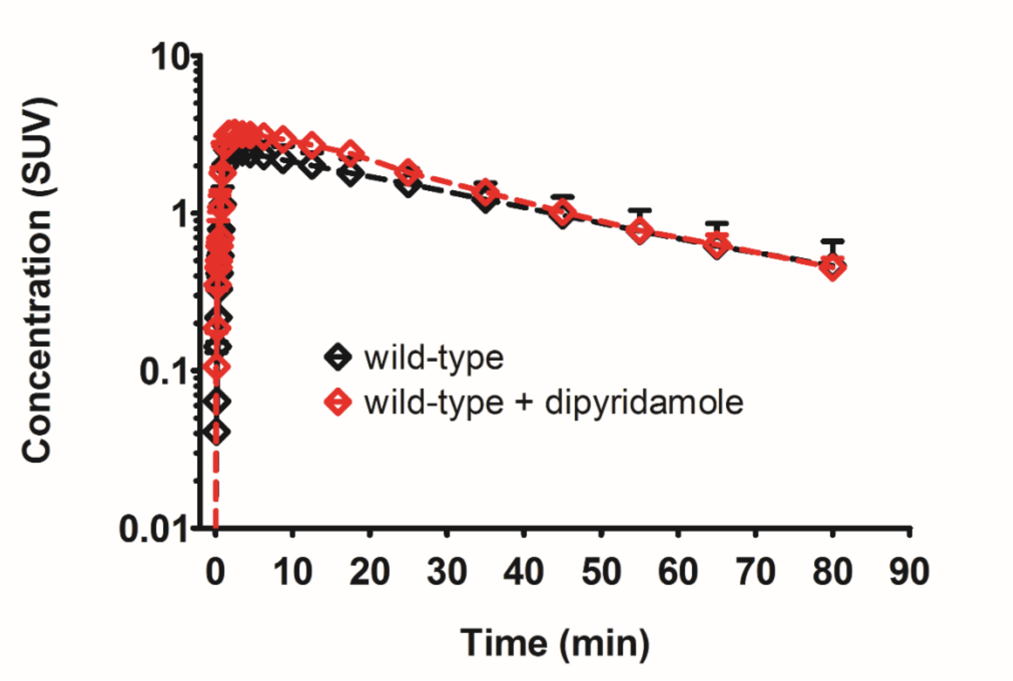

Supplementary Fig 2 Concentration-time curves (mean standardized uptake value, SUV ± SD) in the whole brain of wild-type mice pre-treated at 30 min before PET either with vehicle or with dipyridamole (40 mg/kg, i.p.).

Supplementary References

1. Jewett, DM (1992) A simple synthesis of [^{11}C]methyl triflate. Appl Radiat Isot 43:1383-1385
